# Association of T2/S-RNase With Self-Incompatibility of Japanese Citrus Accessions Examined by Transcriptomic, Phylogenetic, and Genetic Approaches

**DOI:** 10.3389/fpls.2021.638321

**Published:** 2021-02-12

**Authors:** Chitose Honsho, Koichiro Ushijima, Misa Anraku, Shuji Ishimura, Qibin Yu, Frederick G. Gmitter, Takuya Tetsumura

**Affiliations:** ^1^Department of Agricultural and Environmental Sciences, Faculty of Agriculture, University of Miyazaki, Miyazaki, Japan; ^2^Graduate School of Environmental and Life Science, Okayama University, Okayama, Japan; ^3^Citrus Research and Education Center, Institute of Food and Agricultural Sciences, University of Florida, Lake Alfred, FL, United States

**Keywords:** T2 RNase, Citrus, self-incompatibility, RNA-Seq, phylogenetic analysis, S-RNase, S haplotype

## Abstract

Several citrus varieties show gametophytic self-incompatibility (GSI), which can contribute to seedless fruit production in several cultivars. This study investigated the genes regulating this trait through RNA-seq performed using styles collected from the flowers of Japanese citrus cultivars ‘Hyuganatsu,' ‘Tosabuntan,' ‘Hassaku,' ‘Banpeiyu,' and ‘Sweet Spring'. We screened the transcripts of putative T2 RNases, i.e., the protein family including all S-RNases from S-RNase-based GSI plants, and constructed a phylogenetic tree using the screened T2 RNases and S-RNases retrieved from citrus genome databases and a public database. Three major clusters (class I–III) were formed, among which, the class III cluster contained family specific subclusters formed by S-RNase and a citrus-specific cluster monophyletic to the S-RNase clusters. From the citrus class III cluster, six transcripts were consistent with the *S* haplotypes previously determined in Japanese citrus accessions, sharing characteristics such as isoelectric point, extracellular localization, molecular weight, intron number and position, and tissue-specific expression with S-RNases. One T2 RNase gene in self-incompatible Hyuganatsu was significantly down-regulated in the styles of a self-compatible mutant of Hyuganatsu in RNA-seq and qPCR analyses. In addition, the inheritance pattern of some T2 RNase genes was consistent with the pattern of the *S* haplotype in the progeny population of Hyuganatsu and Tosabuntan. As all results supported citrus self-incompatibility being based on S-RNase, we believe that six T2 RNase genes were S-RNases. The homology comparison between the six T2 RNases and S-RNases recently reported in Chinese citrus revealed that three out of six T2 RNases were identical to S-RNases from Chinese citrus. Thus, the other three T2 RNases were finally concluded to be novel citrus S-RNases involved in self-incompatibility.

## Introduction

Self-incompatibility (SI) is a genetic mechanism that causes pistils to reject self-pollen or pollen from close relatives, avoiding a decrease in genetic variability and adaptive potential by self-fertilization (de Nettancourt, 1977). SI is classified into two different genetic forms according to *S* phenotype determination: gametophytic self-incompatibility (GSI) and sporophytic self-incompatibility (SSI), in which incompatible pollen is determined by its own haploid genome or the diploid genome of the plant (sporophyte) that produced it, respectively (Hiscock and McInnis, 2003). GSI is thought to be the most widespread SI system (Franklin-Tong and Franklin, 2003), and to date, two distinct systems of single S-locus GSI have been investigated in detail at the molecular level. The first system was based on the S-RNase system (S-RNase-based GSI) found in Solanaceae, Rosaceae, and Plantaginaceae (Franklin-Tong and Franklin, 2003; Hiscock and McInnis, 2003), while the second was based on PrsS-PrpS interaction, which has so far been found only in *Papaver rhoeas* in the Papaveraceae family (Dresselhaus and Franklin-Tong, 2013).

Considered one of the most important fruit crops worldwide, several *Citrus* varieties showed SI, prevailing mainly in pummelos, mandarins, and their relatives (Yamamoto et al., 2006, 2012). Regarding agricultural citrus production, SI is a leading factor for unstable fruit set, requiring farmers to plant additional pollinizers and/or to perform hand pollination. However, seedless fruits, which are highly favorable in commercial trade, can be produced without special treatments in an SI species when parthenocarpic ability is implemented, because SI reduces the chance of natural seed formation (Yamamoto et al., 1995, 2014).

Soost (1965, 1969) reported that the genetic basis of *Citrus* SI is gametophytic, based on observation of SI in segregating progenies obtained from several mating combinations. Moreover, *Citrus* SI is regulated by co-dominant SI genes at a single multi-allelic locus, including a phenotypical exception of dominant self-fertility (*Sf*) allele (Soost, 1969; Vardi et al., 2000).

Several studies have attempted to elucidate the molecular mechanism and identify the genes representing the *S* determinant in citrus [see the review by Zhang et al. (2018)]. A recent study finally provided detailed evidence of S-RNases being involved in the SI of Chinese varieties of pummelo (*C. maxima* Merr.) and SC of mandarin (*C. reticulata*) by the transition of SI to SC through S-RNase mutation (Liang et al., 2020). However, genes representing *S* haplotypes are still unclear for Japanese SI cultivars.

In Japan, some commercial citrus varieties, including ‘Hyuganatsu' (*C. tamurana* hort. ex Tanaka), ‘Hassaku' (*C. hassaku* hort. ex Tanaka), ‘Banpeiyu' (*C. maxima*), and ‘Tosabuntan' (*C. maxima* or *C. ootachibana* hort. ex Tanaka) showed SI (Miwa, 1951; Yamashita, 1978, 1980; Ngo et al., 2001; Yamamoto et al., 2006; Honsho et al., 2012), which is suggested to contribute to seedlessness in several cultivars (Iwamasa and Oba, 1980; Yamamoto and Tominaga, 2002; Yamamoto et al., 2006). As SI is an important trait both for fruit production and breeding, several studies have been conducted to determine the SI of citrus accessions in Japan (Yamamoto et al., 2006, 2012; Yamamoto, 2014). *S* haplotypes of several Japanese citrus species were determined using *S* allele homozygous plants, which were obtained by bud self-pollination (Kim et al., 2011; Zhou et al., 2018).

Regarding histology, studies on pollen tube growth in the pistil of Japanese SI citrus showed self-pollinated pollen tube growth being aborted at the stigma (Yamashita, 1978) or around the border between stigma and style in ‘Hyuganatsu' (Honsho et al., 2012), at the stigma in ‘Hassaku' (Yamashita, 1980; Ngo et al., 2001), and at the middle style in ‘Tosabuntan' and ‘Banpeiyu' (Ngo et al., 2001). Pollen tube rejection in the pistil tissue is also found in S-RNase-based GSI plants, unlike in other GSI plants of *Papaver* species and some grasses, where pollen rejection occurs at the surface of the stigma (Yang et al., 2008). Autotetraploidized ‘Hyuganatsu' is SC, with the SI reaction in pistils being avoided when its pollen grains pollinated the diploid ‘Hyuganatsu' (Yamashita et al., 1990), and unreduced diploid pollen grains of ‘Nishiuchi Konatsu' (mutant of ‘Hyuganatsu') also overcome SI (Honsho et al., 2012). Polyploidization is a direct cause of SC in the Solanaceae and tribe Pyreae (the genera *Malus* and *Pyrus*) in Rosaceae, which are S-RNase-based GSI plants (Lewis and Modlibowska, 1942; Adachi et al., 2009; Sassa, 2016). Thus, we hypothesized that SI in ‘Hyuganatsu' is regulated by the S-RNase system.

All the known S-RNases are encompassed in the T2 RNase protein family. Previous phylogenetic analysis of T2 RNases indicated that eudicots appear to contain three distinct “classes” of such genes, with S-RNases found exclusively in one of them, Class III (Igic and Kohn, 2001). From evolutionary aspects, the S-RNase-based SI system evolved only once 120 million years ago before the split of the Asteridae and Rosidae (Igic and Kohn, 2001; Wikstrom et al., 2001; Steinbachs and Holsinger, 2002; Vieira et al., 2008). The order Sapindales includes the family Rutaceae, which diversified 80–84 million years ago (Wikstrom et al., 2001). This family includes the genus *Citrus*, which has been proposed to have diversified during the late Miocene epoch (~7 million years ago) in a recent genome analysis (Wu et al., 2018). As *Citrus* appeared after the evolution of S-RNases, it is reasonable to hypothesize that SI in *Citrus* is potentially regulated by S-RNase. In addition to the close phylogenetic relationship, S-RNase genes and proteins generally display a number of shared features, including expression patterns, common intron-exon site patterns, similar isoelectric points (pIs), locus structures, and diversifying selection (Ramanauskas and Igic, 2017). Thus, we previously cloned several style-expressed *S-RNase*-homologous T2 RNase genes from ‘Hyuganatsu' and found that two genes (*CtRNS1* and *CtRNS3*) had common features to S-RNases, such as close phylogenetic position, pistil-specific expression, pI > 8, and a single intron in the genomic structure (Honsho et al., 2019). *CtRNS3* was detected only in the cultivars possessing the *S1* allele determined by Kim et al. (2011), while no specific relationship was found between *CtRNS1* and *S* alleles. However, evidence regarding the association of RNase genes with SI in Japanese *Citrus* is still insufficient. In addition, exploration of *S-RNase*-homologous genes should be expanded to other SI citrus plants as the genetic diversity is essential for the *S* determinant.

In this study, we surveyed the *S-RNase*-homologous genes expressed in styles from several Japanese citrus cultivars by investigating an RNase gene collection in the T2 RNase protein family, encompassing all the known S-RNases, screened from transcriptomes constructed from RNA-seq data. In addition, isolated RNases were further characterized to assess their similarity with S-RNases of other plant species and to investigate their association with SI. Finally, the similarity between S-RNases identified in this study and ones found in Chinese citrus (Liang et al., 2020) was measured to investigate the relationship between S-RNases in Japanese and Chinese citrus.

## Materials and Methods

### Plant Materials

Plant materials used in this study were grown in the field of the University of Miyazaki (Table 1): SI ‘Hyuganatsu' (hereafter HYSI; *C. tamurana*) and its SC mutant (HYSC), ‘Tosabuntan' (TBN; *C. maxima* or *C. ootachibana*), ‘Banpeiyu' (BAN; *C. maxima*), ‘Hassaku' (HSK; *C. hassaku*) and ‘Sweet Spring' (SWS; a hybrid between ‘Ueda Unshiu' satsuma mandarin, i.e., *C. unshiu*, and ‘Hassaku').

**Table 1:** Citrus species used for RNA-seq analysis and genome databases to get T2 RNase sequences and their self-incompatibility status.

**Data source**		**Self-(in)compatibility**	**Prefix on sequence name**	**Database version**	**Reference**	**Database URL**
**Scientific name**	**Common name or cultivar name**					
**RNA-seq**						
*C. tamurana*	‘Hyuganatsu'	SI	HY			
*C. tamurana*	Self-compatible mutant of ‘Hyuganatsu'	SC	HY			
*C. hassaku*	‘Hassaku'	SI	HSK			
*C. maxima or C. ootachibana*	‘Tosabuntan'	SI	TBN			
*C. maxima*	‘Banpeiyu'	SI	BAN			
*C. unshiu* x *C. hassaku*	‘Sweet Spring'	Unknown owing to male sterility	SWS			
**Genome**						
*C. clementina*	Clementine ‘Clemenules' (haploid)	SI	clementine0.9	v0.9	Wu et al. (2014)	https://phytozome.jgi.doe.gov/pz/portal.html
*C. clementina*	Clementine ‘Clemenules' (haploid)	SI	Ciclev10	v1.0	Wu et al. (2014)	https://phytozome.jgi.doe.gov/pz/portal.html
*C. sinensis*	Sweet orange ‘Valencia' (doubled-haploid)	SC	Cs or orange1.1	v2	Xu et al. (2013)	http://citrus.hzau.edu.cn/orange/index.php
*C. medica*	Citron	SI?	Cm	v1	Wang et al. (2017)	http://citrus.hzau.edu.cn/orange/index.php
*C. reticulata*	Mandarin ‘Mangshan'	SC	MSYJ	v1	Wang et al. (2018)	http://citrus.hzau.edu.cn/orange/index.php
*C. ichangensis*	Ichang papeda	SI	Ci	v1	Wang et al. (2017)	http://citrus.hzau.edu.cn/orange/index.php
*C. maxima*	Pummelo (haploid)	SI	Cg	v1	Wang et al. (2017)	http://citrus.hzau.edu.cn/orange/index.php
*C. unshiu*	Satsuma mandarin ‘Miyagawa Wase'	SC	Ciunshiu	Not specified	Shimizu et al. (2017)	http://www.citrusgenome.jp/
*Atalantia buxifolia*	Chinese box orange	Unknown	sb	v1	Wang et al. (2017)	http://citrus.hzau.edu.cn/orange/index.php

*Cultivar name is single-quoted*.

During the flowering season of 2016, styles from TBN, BAN, HSK, and SWS were collected from flowers at the balloon stage. In 2017, additional styles were collected from HYSI and HYSC. Sample collection was duplicated in 2016 and triplicated in 2017 from the same tree on different dates. Young leaves were collected from all the cultivars. Floral and fruit tissues were separately collected from HYSI, i.e., stigma, style, ovary, anther, filament, petal, sepal and pedicel, albedo, flavedo, and juice sac. All samples were quickly frozen in liquid nitrogen immediately after collection and stored at −80°C until further use.

In 2018, HYSI × TBN and reciprocal outcross TBN × HYSI were performed. Seeds were collected and sown in soil to germinate seedlings. Leaves of the parents (HYSI and TBN) were collected for DNA extraction.

### RNA-Sequencing Analysis

#### Library Construction and RNA-seq

Total RNA was extracted from approximately 100 mg of style tissue using NucleoSpin RNA Plus (Macherey-Nagel, Duren, Germany) from all the cultivars according to the manufacturer's protocol. RNA was quantified using a NanoDrop 2000 (ThermoFisher) and Qubit 3.0 Fluorometer (Invitrogen, Carlsbad, CA, USA).

For the style RNA samples collected in 2016, eight Illumina sequence libraries were constructed for each sample using the KAPA Stranded mRNA-Seq Kit (KAPA Biosystems) and sequenced using Illumina HiSeq 4000 to obtain 10M PE100 reads. For the HYSI and HYSC samples obtained in 2017, RNA-seq was performed for six libraries by Novogene to obtain 40M PE150 reads. Samples collected in 2016 and 2017 had two and three replications, respectively.

#### Sequence Processing, Assembly, and Functional Annotation

The RNA-seq reads of HSK, TBN, BAN, and SWS were first processed by Trimmomatic 0.39 with parameters as follows: ILLUMINACLIP:TruSeq2-PE.fa:2:30:10 LEADING:3 TRAILING:3 SLIDINGWINDOW:4:15 MINLEN:36 (Bolger et al., 2014). For ‘Hyuganatsu,' the reads of the HYSI and HYSC samples were combined (HYSI+HYSC) to recover the up/downregulated transcripts between HYSI and HYSC. Those data were preprocessed by Novogene to remove reads containing adapters, N > 10%, and/or low quality (Q ≤ 5) bases (which constituted over 50% of the total bases) before being used for *de novo* assembly. Reads from different replicates of a single cultivar were pooled for *de novo* assembly of the transcriptome. The pooled reads of each cultivar were used as input for a Galaxy-based version of Trinity [www.usegalaxy.com: (Grabherr et al., 2011)] with minimum contig length as 200 and without genome guide mode. The RF parameter for the “–*SS_lib_type*” option was additionally used for the strand-specific libraries of HSK, TBN, BAN, and SWS.

The assembled contigs of the transcriptomes were annotated with Blast2GO version 4.0.2 (BioBam Bioinformatics S.L., Valencia, Spain) using Blastx against the NCBI nr protein database limited to the Viridiplantae with an *E* value cutoff < 1e^−5^; up to 20 hits were retained for every transcript. InterProScan with default parameters was used for the annotation. To assign gene ontology (GO) terms, Enzyme Commission (EC) numbers, and possible descriptions to each contig, the “Mapping and Annotation” function with default parameters was performed with the Blastx and InterProScan results. To evaluate the completeness of the assemblies, we employed Benchmarking Universal Single-Copy Orthologs (BUSCO) v.4.0.5 (Simão et al., 2015) using the reference data set of Viridiplantae odb 10 with 1.0e^−3^ of Blast e-value in OmicsBox 1.2.4 (BioBam Bioinformatics S.L., Valencia, Spain).

#### Screening T2 RNase Genes From Transcriptome and Collection of T2 RNase Including S-RNases From Public Databases

The annotated transcriptomes were filtered for the GO term “ribonuclease T2” or “GO:0033897,” (the GO term assigned to “ribonuclease T2 activity”) to discover putative T2 RNase genes. Obtained sequences were subjected to CD-HIT (Fu et al., 2012) for clustering at 95% similarity, and deduced amino acid sequences were predicted from the longest open reading frame determined by Transdecoder (Haas and Papanicolaou, 2015), cutting off short amino acid sequences (< 200 residues).

T2 RNase genes in some citrus species and their wild relatives (*C. clementina, C. sinensis, C. maxima, C. ichangensis, C. medica, C. unshiu*, and *Atalantia buxifolia*) were obtained from the public genome database (Table 1). Each dataset was searched using the same GO term and number as described above to identify putative T2 RNase genes. Known T2 RNases, including S-RNase of other plant species, were retrieved from our previous study (Honsho et al., 2019) with addition of *CtRNS1* to *3*, which were T2 RNase genes identified in ‘Hyuganatsu' (Honsho et al., 2019). A list of genes is shown in Supplementary Table 1. Their sequences were obtained from the DDBJ/NCBI/EMBL databases.

#### Phylogenetic Analysis

The amino acid sequences of all T2/S-RNase genes were combined for phylogenetic analysis. Alignment was performed using MAFFT (Katoh and Toh, 2008) with the L-INS-i mode provided in GenomeNet (http://www.genomenet.jp/). A phylogenetic tree was constructed by the maximum likelihood method using RAxML software (Stamatakis, 2014) with the “VT+G+I” substitution model, which was determined as the best model by ProtTest (Abascal et al., 2005), with 1,000 bootstraps. The generated tree was drawn using MEGA7 (Kumar et al., 2016).

In the phylogenetic tree, low polymorphic subclusters were found. Their pairwise amino acid sequence identity was calculated as 1-*p*-distance, which was calculated using MEGA7 (Kumar et al., 2016).

#### Characterization of Amino Acid Sequences

Subcellular localization was predicted using DeepLoc-1.0 (http://www.cbs.dtu.dk/services/DeepLoc/) from each amino acid sequence (Armenteros et al., 2017). The pI and molecular weight were determined using an isoelectric point calculator (IPC; http://isoelectric.org/index.html) (Kozlowski, 2016). Six groups were created for data aggregation, i.e., a combination of two datasets for all sequences without the outgroup sequence and only for citrus and three classes in the phylogenetic tree. The outputs were summed separately for each group.

### Exploration of Differential Gene Expression Between HYSI and HYSC

#### Detection of Differential Expressed Genes Between HYSI and HYSC

The reads from style samples of HYSI and HYSC were aligned to the assembled contigs of the HYSI+HYSC transcriptome using Bowtie2 version 2.3.3.1 with default parameters (Langmead and Salzberg, 2012). When a read was mapped to multiple contigs, it was excluded from the counts to retain uniquely mapped reads by excluding reads with “XS:” tag using SAMtools (Li et al., 2009). The number of mapped reads for each contig was counted from the sam files using SAMtools with sort, index, and idxstats commands (Li et al., 2009).

To detect DEGs between HYSI and HYSC samples (three replicates for each), the DESeq2 package (Love et al., 2014) was used on the count data in R 3.4.1 (R Core Team, 2016). The threshold value of false discovery date (FDR) for DEGs was set to 0.05.

#### Quantitative Real-Time PCR (qRT-PCR) Analysis

Styles of HYSI and HYSC were used. Total RNA was extracted using the NucleoSpin RNA Plus kit (Macherey-Nagel) mentioned above and treated with TURBO DNA-free™ Kit (Ambion) to remove contaminating DNA. To synthesize single-strand cDNA, we used an Affinity Script qPCR cDNA synthesis kit (Agilent) according to the manufacturer's instructions. Brilliant III Ultra-Fast SYBR Green qPCR Master Mix (Agilent Technologies, Santa Clara, US) was used to generate a reaction mixture with gene-specific primers (Supplementary Table 2) designed by Primer3 software (Untergasser et al., 2012) for performing qRT-PCR. Reactions were performed as follows: 95°C for 3 min, followed by 40 cycles of 95°C for 5 s and 60°C for 10 s. Three biological replicate samples were used. Citrus *beta actin* was used to normalize the mRNA levels. Seven T2 RNase transcripts (HY15217_c0_g1_i1, HY7198_c0_g1_i2, HY11404_c0_g1_i5, HY9350_c0_g2_i1, HY11692_c0_g2_i2, HY16290_c1_g3_i1, and HY 8523_c0_g1_i1) were selected from a total of nine T2 RNase transcripts obtained from the HYSI+HYSC transcriptome because two excluded transcripts (HY11404_c0_g1_i12 and HY11404_c0_g1_i18) were isoforms of HY11404_c0_g1_i5 and showed very high similarity to the other two sequences. Quantitative variation was analyzed using the formula 2^−ΔΔCT^ (Livak and Schmittgen, 2001).

### Sanger Sequencing of T2 RNase Genes

**Figure 1:**
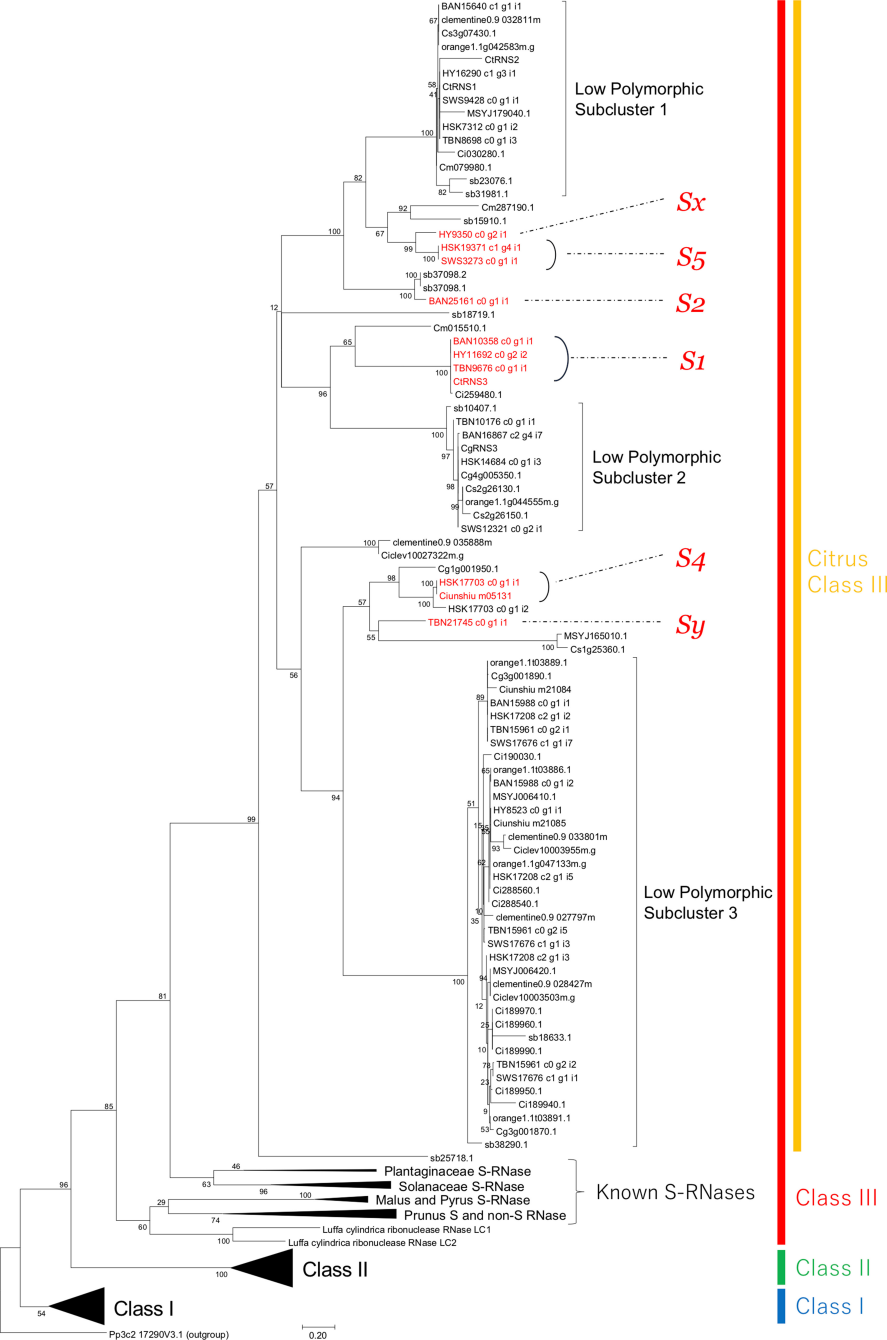
Phylogenetic tree of T2 RNases constructed by maximum likelihood method. Clusters other than citrus were compressed and expressed by solid triangles. Possible citrus S-RNases inferred from the correspondence to determined *S* haplotypes were shown in red. The numbers nearby branches indicated bootstrap values from 1,000 replications.

The genome sequences of the selected eight citrus T2 RNase transcripts (HY9350_c0_g2_i1, HY11692_c0_g2_i2, HY16290_c1_g3_i1, HY 8523_c0_g1_i1, HSK_1703 c0 g1 i1, HSK19371_c1_g4_i1, TBN21745_c0_g1_i1, and BAN25161_c0_g1_i1) in class III of the phylogenetic tree [Figure 1; Igic and Kohn (2001)] were determined. The whole genomic region of each gene, including exons and introns, was amplified by PrimeSTAR® GXL DNA Polymerase (TaKaRa Bio) with a gene-specific primer set (Supplementary Table 2) and a DNA template of cultivars from which the target gene was originally obtained from RNA-seq. PCR amplicons were subcloned using pGEM®-T Easy Vector System (Promega) and *E. coli* DH5α competent cells. The target sequences were read using the Sanger method. For each sequence, three clones were sequenced. The exon/intron structure of the sequence-determined T2 RNase genes was identified based on the alignments of their coding region and the corresponding whole gene sequences using Gene Structure Display Server 2.0 (http://gsds.cbi.pku.edu.cn/) (Hu et al., 2015). Amino acid sequences of the coding region were aligned with MAFFT using the L-INS-i option in GenomeNet (http://www.genomenet.jp/). The alignment sequence was drawn using JalView (Waterhouse et al., 2009).

### Investigation of Tissue-Specific Expression by Semi-quantitative RT-PCR

Tissue-specific expression was investigated by semi-quantitative RT-PCR considering tissues of style, stigma, ovary, anther and pollen, filament, sepal, and petal from flowers, and albedo, flavedo, and juice sac from mature fruits. RNA was extracted with a NucleoSpin RNA Plus kit (Macherey-Nagel) following the manufacturer's instructions. Reverse transcription was performed with 1 μg of total RNA and ReverTra Ace® qPCR RT Master Mix with gDNA Remover (Toyobo, Osaka, Japan) in a 20 μL reaction volume, followed by 10-fold dilution with TE buffer. Semi-quantitative RT-PCR was conducted in a volume of 20 μL containing 2 μL of diluted cDNA solution, 0.5 μM of each primer (Supplementary Table 2), and 1 × EmeraldAmp® MAX PCR Master Mix (TaKaRa Bio, Shiga, Japan). PCRs were performed with a T-100 thermal cycler (Bio-Rad) with the following steps: 94°C for 10 min followed by 32 cycles of 94°C for 16 s, 55°C for 20 s, 72°C for 20 s, and a final step at 72°C for 10 min. The amplified products were electrophoresed using 1% agarose gels. They were stained with EtBr, and fragments were visualized under UV exposure.

### Inheritance Analysis of *T2 RNase*

The number of seedlings was 81 and 95 for HYSI × TBN and TBN × HYSI, respectively. DNA was extracted from the leaves of the progeny and their parents (HYSI and TBN) by the CTAB method (Doyle and Doyle, 1987). Six *T2 RNase* loci (HY16290, HY11692, HY8523, HY9350, TBN10176, and TBN21745), which were positioned in the class III cluster from the phylogenetic tree (Figure 1), were amplified by PCR using EmeraldAmp (TaKaRa). Primer sequences were listed in Supplementary Table 2. The PCR reaction was carried out at 95°C for 1 min as the initial denaturation, followed by 30 cycles of 95°C for 30 s, 57°C for 30 s, and 72°C for 30 s, and 72°C for 2 min as final extension. Amplicons were electrophoresed on a 1% agarose gel and stained with EtBr. Fragments on the gel were visualized by exposure to UV radiation. The number of individuals with or without *T2 RNases* (present/absent) was tested by chi-square test for their fitness of observed ratio to the expected ratio of 1:1 and 3:1.

### Correspondence of Isolated T2 RNase to S-RNases

T2 RNases obtained in this study and possibly involved in SI, and 15 *S-RNase*s (*S1* to *S14* and *Sm*^*R*^*-RNase*s: accession numbers are MN652897 to MN652910, and MN652912) reported by Liang et al. (2020) were compared. The alignment and its visualization were as described above. Based on the alignment sequences, pairwise numbers of different sites and *p-distances* were calculated using MEGA7 (Kumar et al., 2016).

## Results

### RNA-seq, *De novo* Assembly, and Annotation

RNA-seq and *de novo* assembly results are summarized in Tables 2, 3, respectively. The mean number of raw pair reads per library in HYSI+HYSC and other varieties was 25,296,882 and 7,846,791, respectively (Table 2). The overall effective ratio, which was the ratio of trimmed reads to raw reads, was 95.57% (Table 2). After *de novo* assembly, the number of contigs generated spanned from 72,312 (SWS) to 203,776 (HYSI+HYSC) with a maximum length of approximately 15 kbp (Table 3). BUSCO analysis indicated that over 85% of contigs covered complete sequences of core collection, including single- and multiple-copies, although ‘Hyuganatsu' resulted in a higher percentage (63.0%) of complete and duplicated genes than the others (36.3% on average), probably because input contigs of HYSI+HYSC were much higher than other cultivars (Table 3). After annotation, over 80% of contigs were blasted and ~70% of contigs were annotated with one or more GO terms (Table 3). Detailed annotation results are all provided in Supplementary Table 3.

**Table 2:** Results of sequencing and quality filtering of reads.

**Sample name**	**Data type**	**Number of raw read pairs**	**Number of trimmed read pairs**	**Effective rate (%)**^**z**^	**Accession number**^**y**^
Hyuganatsu HYSI style rep 1	Pair-end 150 bp	26,743,939	25,869,359	96.73	DRR237215
Hyuganatsu HYSI style rep 2	Pair-end 150 bp	27,863,139	26,955,874	96.74	DRR237216
Hyuganatsu HYSI style rep 3	Pair-end 150 bp	25,582,779	24,771,172	96.83	DRR237217
Hyuganatsu HYSC style rep 1	Pair-end 150 bp	21,627,816	20,765,629	96.01	DRR237220
Hyuganatsu HYSC style rep 2	Pair-end 150 bp	27,084,791	26,077,432	96.28	DRR237221
Hyuganatsu HYSC style rep 3	Pair-end 150 bp	22,878,825	22,028,018	96.28	DRR237222
Tosabuntan style rep 1	Pair-end 100 bp	8,188,485	7,745,169	94.59	DRR237225
Tosabuntan style rep 2	Pair-end 100 bp	8,064,045	7,612,201	94.40	DRR237226
Banpeiyu style rep 1	Pair-end 100 bp	7,726,017	7,278,966	94.21	DRR237227
Banpeiyu style rep 2	Pair-end 100 bp	8,537,110	8,090,527	94.77	DRR237228
Hassaku style rep 1	Pair-end 100 bp	7,265,353	6,911,044	95.12	DRR237229
Hassaku style rep 2	Pair-end 100 bp	7,462,773	7,135,460	95.61	DRR237230
Sweet Spring style rep 1	Pair-end 100 bp	7,525,801	7,176,928	95.36	DRR237231
Sweet Spring style rep 2	Pair-end 100 bp	8,004,741	7,606,753	95.03	DRR237232

z Effective Rate (%): (Clean reads/Raw reads)*100.

y *DDBJ DRA Run*.

**Table 3:** Summary of *de novo* assembly and functional annotation of style transcriptomes in citrus.

		**Hyuganatsu**		**Hassaku**		**Tosabuntan**		**Banpeiyu**		**Sweet Spring**	
Assembly	Contig count	203,776		75,602		81,579		88,033		72,312	
	Maximum	15,266		15,696		15,707		12,253		15,787	
	Minimum	201		201		201		201		201	
	Average	1,499.6		1,194.8		1,258.9		1,266.9		1,226.5	
	N50	2,406		1,872		2,008		2,001		1,942	
			%		%		%		%		%
BUSCO	Complete (C) and single-copy (S)	104	24.2	225	52.3	202	47.0	203	47.2	248	57.7
	Complete (C) and duplicated (D)	271	63.0	144	33.5	172	40.0	181	42.1	127	29.5
	Fragmented	46	10.7	31	7.2	34	7.9	30	7.0	33	7.7
	Missing	9	2.1	30	7.0	22	5.1	16	3.7	22	5.1
Annotation	Blasted	166,866	81.9	63,004	83.3	66,572	81.6	72,263	82.1	60,248	83.3
	No-Blast	36,910	18.1	12,598	16.7	15,007	18.4	15,770	17.9	12,064	16.7
	InterPro hit	136,511	67.0	52,327	69.2	57,300	70.2	61,741	70.1	50,169	69.4
	Mapped	141,541	69.5	52,677	69.7	53,805	66.0	60,631	68.9	50,512	69.9
	Annotated	135,327	66.4	51,404	68.0	52,974	64.9	59,213	67.3	49,477	68.4

The putative *T2 RNase* genes were screened from the annotated transcripts constructed from RNA-seq reads based on the GO term and number, finding 9, 14, 9, 9, and 9 *T2 RNase* genes in HYSI+HYSC, HSK, TBN, BAN, and SWS, respectively (Table 4). Several genes in the citrus genomic data retrieved from the public domain were annotated with T2 RNase, ranging from 6 (*C. clementina* (v.1.0), *C. reticulata*, and *C. unshiu*) to 16 (*C. sinensis*) (Table 4).

**Table 4:** Number of T2 *RNase* genes in genome or transcripts in style tissue categorized into class I to III.

**Data source**		**Number of T2 RNase**				**Genes or transcripts without low polymorphic subcluster in class III**
**Scientific name**	**Common name or cultivar name**	**Class I**	**Class II**	**Class III**	**Total**	
**RNA-seq**						
*C. tamurana*	‘Hyuganatsu'^z^	2	3	4	9	2
*C. hassaku*	‘Hassaku'	1	5	8	14	2 (3)^y^
*C. maxima or C. ootachibana*	‘Tosabuntan'	1	1	7	9	2
*C. maxima*	‘Banpeiyu'	1	2	6	9	2
*C. unshiu* x *C. hassaku*	‘Sweet Spring'	2	1	6	9	1
**Genome**						
*C. clementina* v0.9	Clementine ‘Clemenules' (haploid)	2	1	5	8	1
*C. clementina* v1.0	Clementine ‘Clemenules' (haploid)	2	1	3	6	1
*C. sinensis*	Sweet orange ‘Valencia' (doubled-haploid)	4	2	10	16	1
*C. medica*	Citron	2	3	3	8	2
*C. reticulata*	Mandarin ‘Mangshan'	1	1	4	6	1
*C. ichangensis*	Ichang papeda	1	4	10	15	1
*C. maxima*	Pummelo (haploid)	2	2	4	8	1
*C. unshiu*	Satsuma mandarin ‘Miyagawa Wase'	2	1	3	6	1
*Atalantia buxifolia*	Chinese box orange	2	3	10	15	5

z Cultivar name is single-quoted.

y *Number in a parenthesis is original number in the phylogenetic tree. One sequence was expected to be generated by incorrect assemble*.

### Phylogenetic Analysis

The maximum likelihood tree revealed three distinct clusters, which corresponded to the classes I–III defined by Igic and Kohn (2001) (Figure 1). The overall topology of the tree was identical to a tree in our previous study (Honsho et al., 2019); S-RNases of Rosaceae, Solanaceae, and Plantaginaceae formed family specific clusters in the class III cluster (Figure 1). Several citrus T2 RNases obtained from RNA-seq data and public genome databases were included in a cluster monophyletic to the S-RNases clusters in class III. The number of T2 RNases belonging to each cluster for each citrus species is indicated in Table 4. Among the class III citrus clusters, there were three subclusters formed by low polymorphic sequences (Figure 1). The averages of pairwise identities of low polymorphic subclusters (LPSCs) 1, 2, and 3 were 91.4, 96.7, and 89.4%, respectively. Our previously isolated T2 RNase sequences, CtRNS1 and CtRNS3, showed a complete match with some transcripts, i.e., CtRNS1 was identical to HY16290_c1_g3_i1, SWS9428_c0_g1_i1, HSK7312_c0_g1_i2, and TBN8698_c0_g1_i3, and CtRNS3 was HY11692_c0_g2_i2, BAN10358_c0_g1_i1, and TBN9676_c0_g1_i1. (Figure 1, Honsho et al., 2019). As all the S-RNases in other S-RNase-based GSI plants are positioned in class III (Igic and Kohn, 2001), we focused on the transcripts in the citrus class III cluster for further characterization, expression analysis, inheritance analysis and similarity searches with S-RNases of Chinese citrus cultivars to investigate their association with the SI reaction.

### Characteristics of T2 RNases From Amino Acid Sequences

Subcellular localization and pI of proteins from each amino acid sequence were predicted for each class in the phylogenetic tree. The average pI for the data including all members was 5.166, 5.836, and 8.795 for class I, II, and III, respectively, while the average pI for the data limited to citrus was 5.218, 5.705, and 8.771 for classes I, II, and III, respectively (Supplementary Figure 1). The majority of predicted subcellular localization was extracellular in class I, II, and III, i.e., 100, 72, and 87% for the whole dataset and 100, 71, and 84% for citrus subset data, respectively. The molecular weight of most T2 RNases was distributed around 25 kD, regardless of the class.

### Detection of Differentially Expressed Genes (DEGs) and Validation of Gene Expression by qRT-PCR

DEG analysis results showed 40 and 39 transcripts up and downregulated, respectively, comparing HYSC to HYSI (Supplementary Table 4). One downregulated transcript (HY9350_c0_g2_i1; hereafter HY9350) was annotated as T2 RNase, and was positioned in the class III cluster (Figure 1).

**Figure 2:**
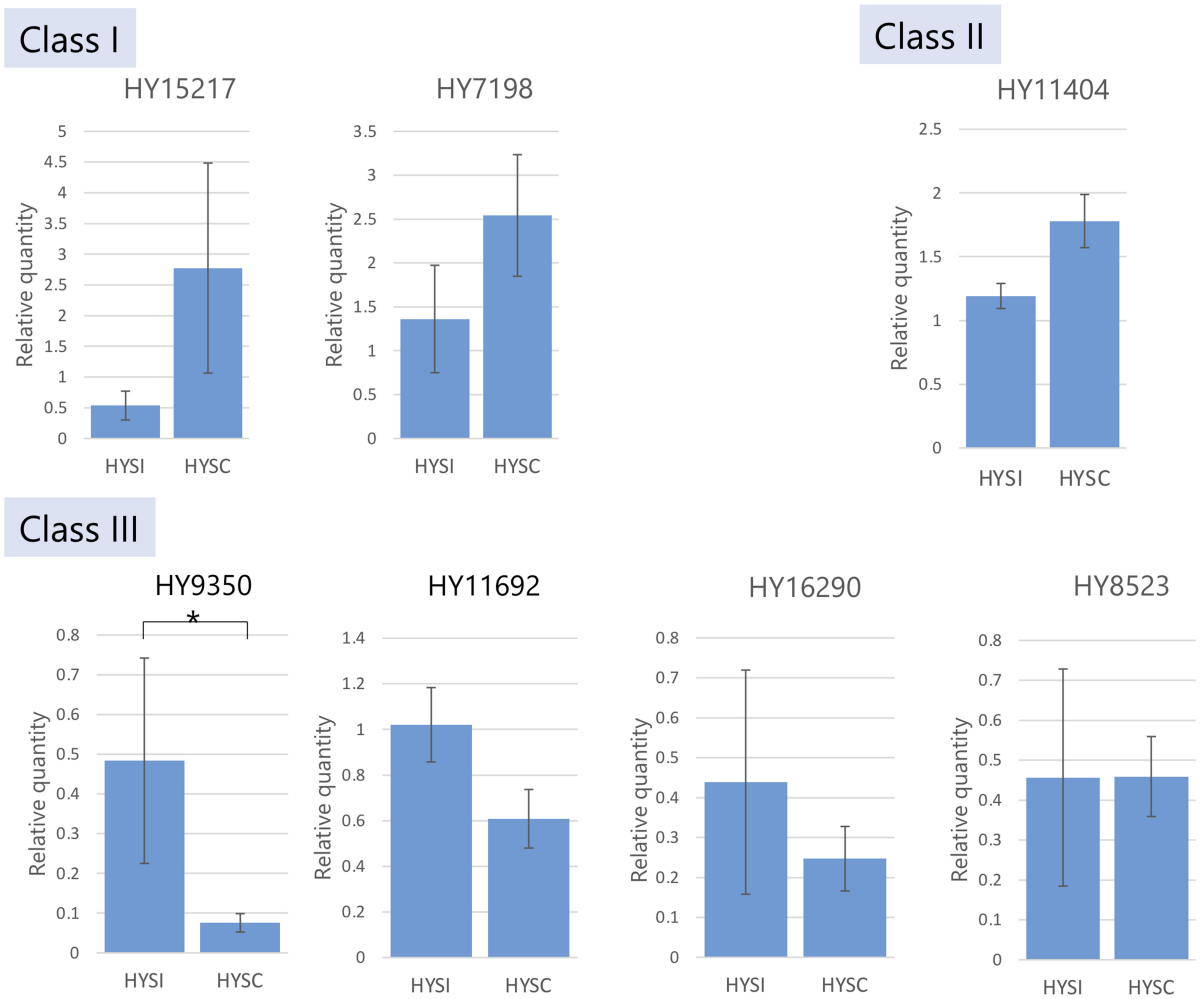
RT-qPCR analysis for gene expressions of seven *T2 RNases* in HYSI and HYSC style tissues. Vertical bars indicate standard error and *denoted significant differences at *P* ≤ 0.05.

Relative gene expression levels for T2 RNase genes were validated by qPCR in HYSI and HYSC. Among the seven T2 RNase genes tested, one gene (HY9350), detected as a downregulated DEG (Supplementary Table 4), showed significantly lower expression in HYSC than in HYSI (Figure 2). Other gene expressions did not show a significant difference between HYSI and HYSC.

### Gene Sequence and Structure of *T2 RNase*

The putative gene structures of the predicted T2 RNase genes along with intron/exon distribution patterns were shown in Supplementary Figure 2. According to the predicted structures, all T2 RNases in the class III cluster had one intron, positioned in the non-conserved hypervariable (HV) region as in other S-RNase-based GSI plants. From the alignment data, two histidine sites, which are essential for RNase activity (Kawata et al., 1990; Parry et al., 1997; MacIntosh, 2011), were conserved in all sequences of predicted T2 RNase (Supplementary Figure 2). The intron length ranged from 87 to 374 bp. Sequencing revealed that HY8523_c0_g1_i1 was, in fact, a compound of two similar but different sequences. They were named HY8523a (accession number; LC575205) and HY8523b (LC575206), and both had one intron in the same position as other class III T2 RNase genes (Supplementary Figure 2). HY8523a (143 amino acids) was shorter than other sequences (Supplementary Figure 2).

### Expression Analysis of *T2 RNase* in Different Tissues

**Figure 3:**
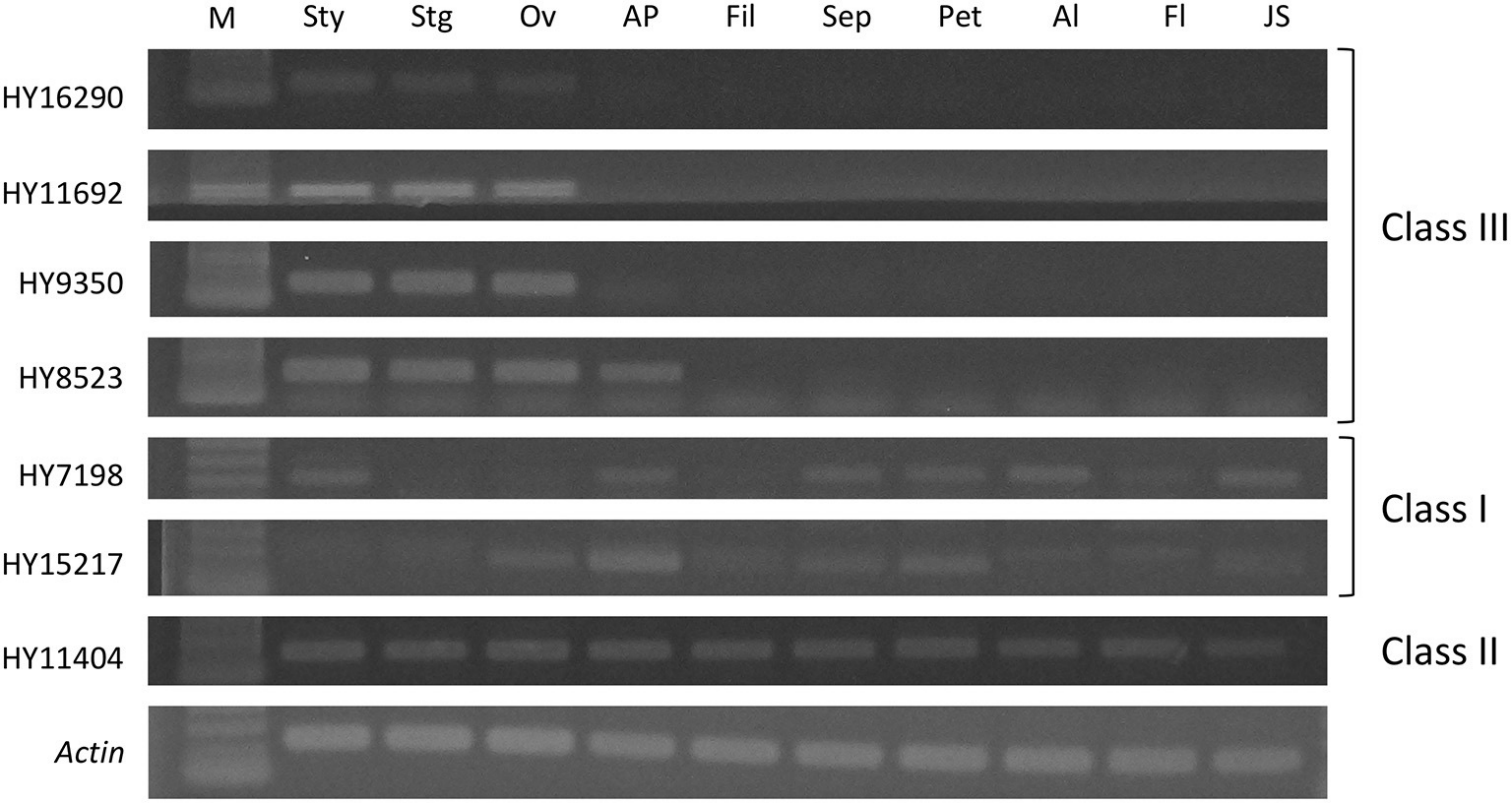
Expression analysis of seven *T2 RNase* genes in different tissues from flowers and fruits of Hyuganatsu by semi-quantitative RT-PCR. Sty, style; Stg, stigma; Ov, ovary; AP, Anther and Pollen; Fil, Filament; Sep, Sepals; Pet, Petals; Al, Albedo; Fl, Flavedo; JS, Juice sac; M, 100 bp molecular marker.

HY16290, HY11692, and HY9350 in class III were expressed in stigma, style, and ovary tissues, indicating that their expression was specific to female organs (Figure 3). HY8523, which was located at a low polymorphic subcluster in the class III cluster (Figure 1), was expressed not only in female tissue but also in anther and pollen tissue. For genes in class I and II, gene expression was not specific to the female organ.

### Inheritance Mode of *T2 RNase* in the HYSI × TBN and TBN × HYSI Populations

**Figure 4:**
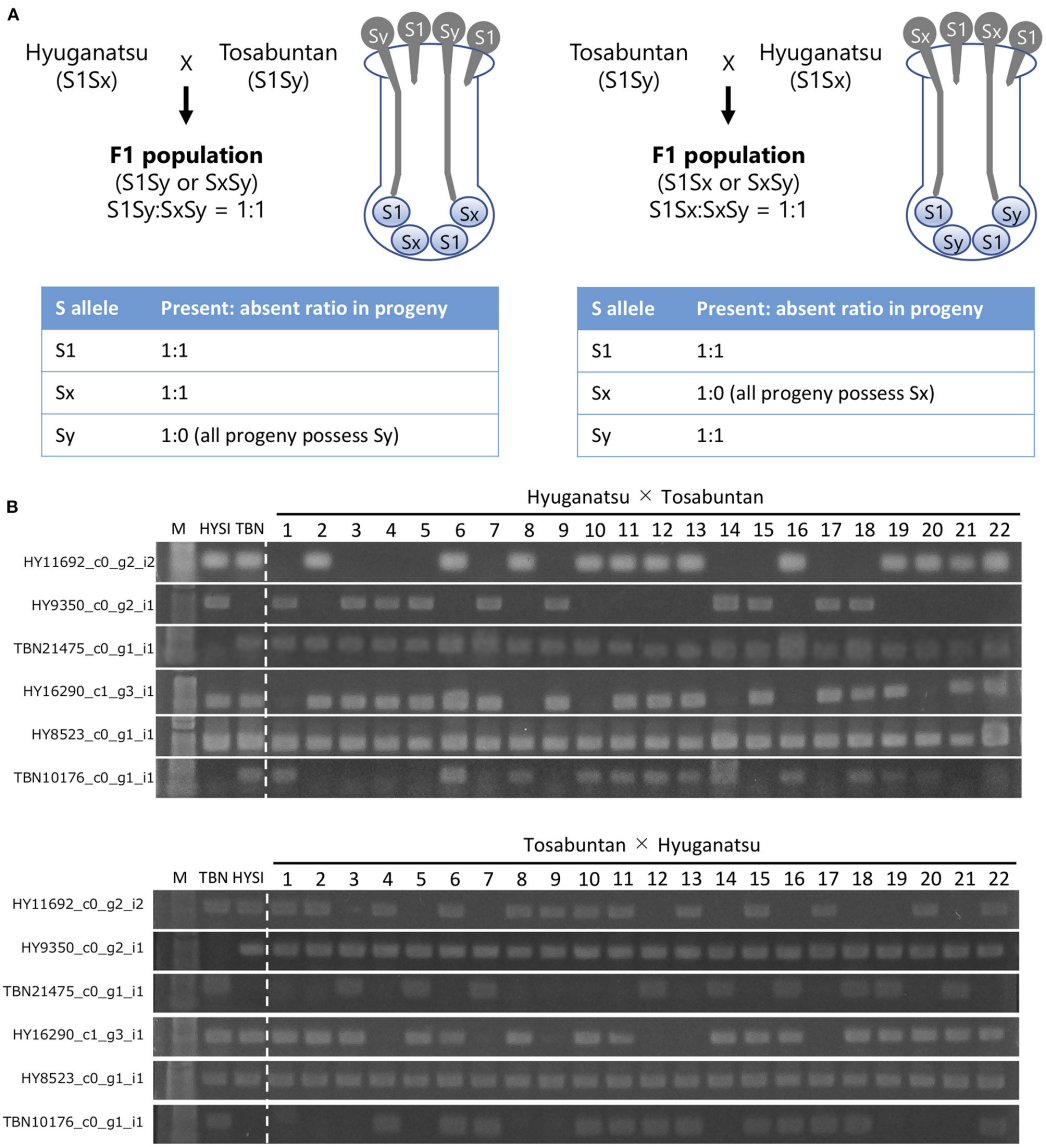
Schematic drawing of the inheritance pattern of *S* allele in semi-compatible pollination **(A)** and inheritance of selected *T2 RNase*s in the progenies of the reciprocal crosses of Hyuganatsu (HYSI) and Tosabuntan (TBN) **(B)**. When semi-compatible HYSI (*S1Sx*) and TBN (*S1Sy*) are outcrossed, in the HYSI × TBN progeny population, only *S1Sy* and *SxSy* can be found at same ratio (1:1) because pollen grains with *S1* allele from Tosabuntan are completely rejected by self-incompatible reaction. An expected ratio of present/absent for *S1, Sx* and *Sy* allele is 1:1, 1:1, and 1:0, respectively. In the reciprocal TBN × HYSI population, *S1Sx* and *SxSy* appear as 1:1 ratio and expected ratio of present/absent for *S1, Sx* and *Sy* allele is 1:1, 1:0, and 1:1, respectively.

HYSI possessed HY16290, HY11692, HY8523, and HY9350, while TBN had HY16290, HY11692, HY8523, TBN10176, and TBN21475 (Figure 4; Table 5). HYSI and TBN share one *S* haplotype, but are still cross-compatible. If a *T2 RNase* associates with SI, its inheritance will not follow the Mendelian law of independent assortment, resulting in a distorted segregation ratio (Figure 4). In the χ^2^ goodness of fit test, HY16290 fitted to a 3:1 (present:absent) ratio at *P* = 0.05, while HY11692, HY9350, and TBN10176 fitted to a 1:1 HYSI × TBN population (Table 5). In addition, either HY11692 or HY9350 and either HY11692 or TBN21475 were inherited in the HYSI × TBN and TBN × HYSI population, respectively, indicating that they were located at the same locus. Contrastingly, all individuals of the progeny population had HY8523 and TBN21475. Thus, the proportion did not fit to neither 1:1 nor 3:1. In the TBN × HYSI population, HY16290 fitted to 3:1, while HY11692, TBN10176, and TBN21475 fitted to 1:1 (Table 5). All individuals in the progeny population had HY8523 and HY9350; thus, the number of progenies fitted either 3:1 or 1:1 of the expected ratios.

**Table 5:** Inheritance and segregation of T2 *RNase* genes in two populations of Hyuganatsu and Tosabuntan reciprocal crosses.

	**Hyuganatsu**	**Tosabuntan**	**Hyuganatsu** **×** **Tosabuntan population**				**Tosabuntan** **×** **Hyuganatsu population**			
**T2 RNase**			**Present**	**Absent**	***P*** **value (χ**^**2**^ **test)**		**Present**	**Absent**	***P*** **value (χ**^**2**^ **test)**	
					**1:1**	**3:1**			**1:1**	**3:1**
HY16290	+	+	58	23	0.0001007	0.4804	71	22	3.75 × 10^−7^	0.7647
HY11692	+	+	43	38	0.5785	5.25 × 10^−6^	42	51	0.3507	3.02 × 10^−11^
HY8523	+	+	81	0	<2.2 × 10^−16^	2.04 × 10^−7^	93	0	<2.2 × 10^−16^	2.58 × 10^−8^
HY9350	+	–	38	43	0.5785	5.29 × 10^−9^	93	0	<2.2 × 10^−16^	2.58 × 10^−8^
TBN10176	–	+	39	42	0.7389	2.39 × 10^−8^	38	55	0.07793	2.89 × 10^−14^
TBN21475	–	+	81	0	<2.2 × 10^−16^	2.04 × 10^−7^	51	42	0.3507	7.118 × 10^−6^

### Homology Between T2 RNases and S-RNases, and Correspondence to *S* Haplotypes

**Figure 5:**
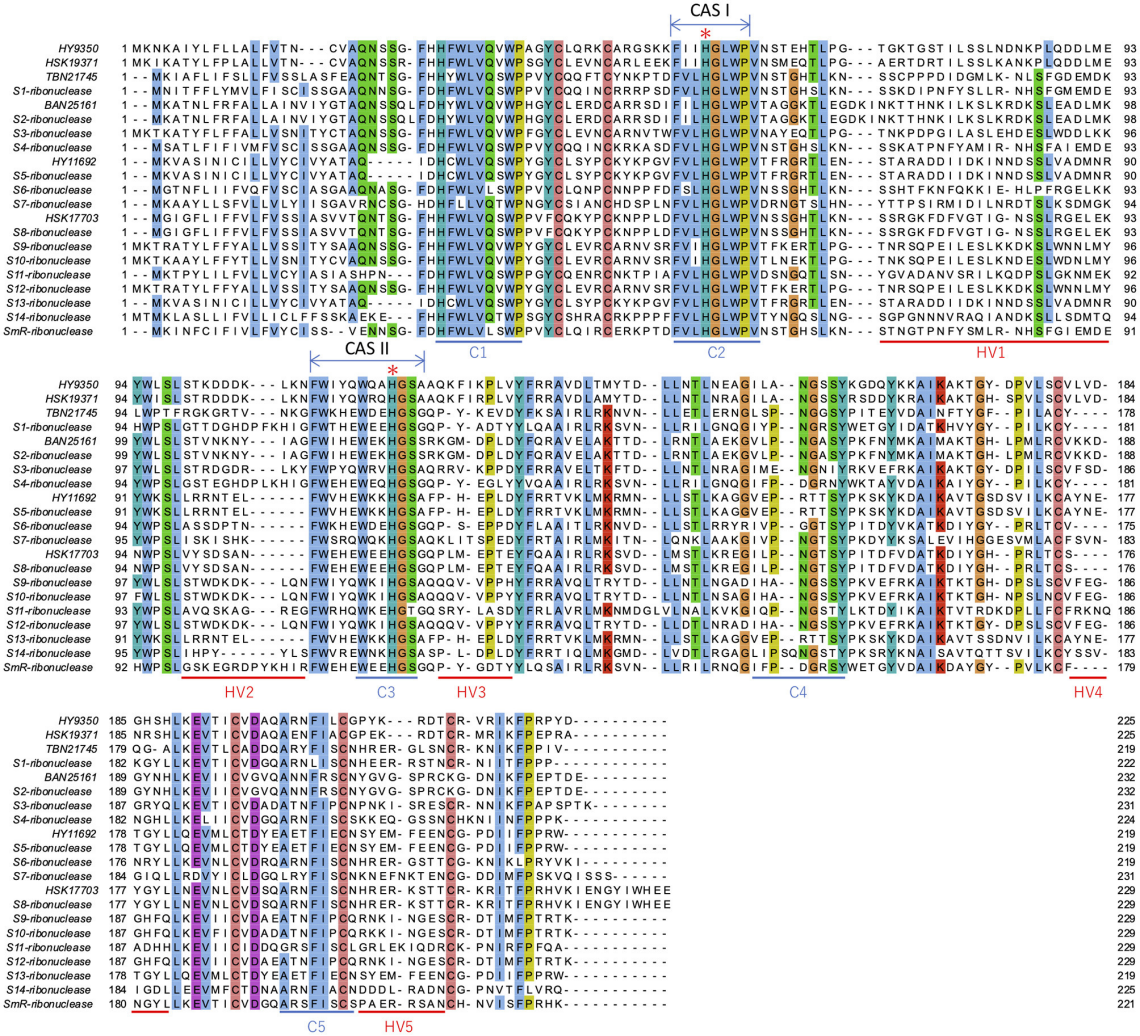
Alignment of T2 RNases from this study and reported S-RNases. The sites with over 70% identity are colored. Conserved region (C1 to C5) and hyper variable region (HV1 to HV5) are indicated below the sequences. Additionally, the two conserved regions (CAS I and II) are indicated above the sequences. Two histidine residues essential for RNase activities in CAS I and II (Kawata et al., 1990; Parry et al., 1997) are indicated by asterisks. Note that BAN25161 and S2-ribonuclease, HY11692 and S5-ribonuclease, and HSK17703 and S8-ribonuclease are completely identical.

The homology between *T2 RNases*, which were expected to be involved in SI in this study, and S-RNases reported by Liang et al. (2020) were investigated. HY11692, BAN25161, and HSK17703 were completely identical to *S5, S2*, and *S8* ribonuclease, respectively (Supplementary Table 5). The overall identity was 46.2% (*p*-distance: 0.54). Five conserved regions (C1 to 5) and five HV regions (HV1 to 5) as well as two conserved histidine residues essential for RNase were found in the alignment (Figure 5), suggesting that they possibly have S-RNase activities. The association between *T2 RNase*s in this study, *S-RNase*s by Liang et al. (2020), and *S* haplotypes determined by Kim et al. (2011) and Zhou et al. (2018) are summarized in Table 6. As it was found that three *T2 RNases* were non-redundant to the *S-RNases* of Liang et al. (2020), we propose that HY9350, TBN21745, and HSK19371 are assigned as *S15*-, *S16*-, and *S17-RNase*, respectively (Table 5).

**Table 6:** Association between *S-RNase*s obtained in this study, reported *S-RNase*s, and *S* haplotypes.

**Possible *S-RNase* identified in this study**	**Corresponding *S-RNase*s presented by Liang et al. (2020)**	**Corresponding *S* haplotypes determined by Kim et al. (2011) and Zhou et al. (2018)**	**Proposed numbering of *S* haplotype and *S-RNase***
HY9350	-	-	*S15*
HY11692	*S5*-ribonuclease	*S1*	*S5*
TBN21745	-	-	*S16*
HSK19371	-	*S5*	*S17*
BAN25161	*S2*-ribonuclease	*S2*	*S2*
HSK17703	*S8*-ribonuclease	*S4*	*S8*

## Discussion

### A Phylogenetic Tree Gave a Consistent Correspondence Between T2 RNases and *S* Haplotypes

S-RNase-based GSI is widely found in core eudicots, including the highly divergent Asterid and Rosid lineages (Igic and Kohn, 2001; Steinbachs and Holsinger, 2002; Roalson and McCubbin, 2003). The shared use of S-RNases implies that the genes underlying RNase-based SI may be molecular homologs (orthologs), remarkably conserved remnants of a trait that arose in a common ancestor over 100 million years ago, whose descendants include nearly three-quarters of plant species (Xue et al., 1996; Igic and Kohn, 2001; Steinbachs and Holsinger, 2002; Ramanauskas and Igic, 2017). Therefore, phylogenetic analysis could be useful for finding *S-RNase*-homologous genes and to predict their functions from amino acid and nucleotide sequence features.

In the citrus class III cluster (Figure 1), three LPSCs-contained numerous citrus T2 RNases, and intra-subcluster amino acid similarity was high (89.4–96.7%) compared to the intra-specific S-RNase similarity in *Prunus* and *Nicotiana*, roughly spanning from 60 to 80% (Kheyr-Pour et al., 1990; Sassa et al., 1996; Ushijima et al., 1998; Yamane et al., 1999). Thus, it is suggested that sequences in LPSCs do not have adequate diversity to be established as *S* determinants. In addition, among the sequences in LPSC, HY11692 (LPSC 1), TBN10176 (LPSC 2), and HY8523 (LPSC 3) were shown as not associated with SI by inheritance analysis, which revealed that they were located on different chromosomes from the *S* locus (see later discussion). S-RNase-like genes (non-S-RNase), which are phylogenetically close to S-RNase but not involved in self-incompatible reactions, have been found in other S-RNase-based GSI plants, such as *Prunus* species (Yamane et al., 2003; Aguiar et al., 2015; Morimoto et al., 2015), *Petunia inflata* (Lee et al., 1992), and *Nicotiana alata* (Kuroda et al., 1994), although they were not mentioned in the S-RNase study of Chinese citrus (Liang et al., 2020). It has been suggested that S-RNase has experienced multiple duplications to diverge S-RNase and non-S-RNase during the evolution of Rosaceae (Morimoto et al., 2015). Thus, the function of genes in LPSC might have different role(s) from other genes in the class III cluster, although they are expected to share the same ancestral sequence.

Excluding LPSCs, the remaining number of *T2 RNases* in class III ranged from 0 to 3 for every citrus species (Table 4). The numbers except for HSK seem *S* determinant (S-RNase) because diploid SI plants, SC plants, and haploid or doubled haploid plants have 2, 1, and 1 gene/transcript(s), respectively. In HSK, three *S-RNase*-homologous genes seem excessive as the *S* gene. However, two of them, HSK17703_c0_g1_i1 and HSK17703_c0_g1_i2, were output as an isoform by *de novo* assembly program. A cloning trial for the genome sequence of HSK was successful for HSK17703_c0_g1_i1, but not for HSK17703_c0_g1_i2, suggesting that the latter was generated by an incorrect assembly. In addition, it was found that Ciunshiu m05131 in satsuma mandarin genome had the same sequence as HSK17703_c0_g1_i1 (Figure 1). Thus, it is concluded that only HSK17703_c0_g1_i1 is present in the HSK genome, and HSK had two genes remaining in the class III cluster by excluding LPSCs and HSK17703_c0_g1_i2.

Kim et al. (2011) and Zhou et al. (2018) determined *S* haplotypes of HY, TBN, BAN, HSK, SWS, and satsuma mandarins as *S1S?, S1S?, S1S2, S4S5, SfS5*, and *SfS4*, respectively, where *Sf* is the self-compatible haplotype and *S?* is an undetermined haplotype. Because HY is cross-compatible with TBN (Ogata et al., 2008), their unknown *S* haplotypes are not the same; therefore, *S* haplotypes were here assigned as *S1Sx* and *S1Sy* (*x* is not equal to *y* and they are not 1, 2, 4, or 5) for HY and TBN, respectively. Considering the sharing status of *S* haplotypes, a consistent correspondence was found between determined *S* haplotypes and T2 RNases within the class III cluster excluding LPSC (Figure 1). Their correspondences were as follows, *S1*: BAN10358_c0_g1_i1, HY11692_c0_g2_i2 and TBN9676_c0_g1_i1; *S2*: BAN25161_c0_g1_i1; *S4*: HSK17703_c0_g1_i1 and Ciunshiu_m05131; *S5*: HSK19371_c0_g4_i1 and SWS3273_c0_g1_i1; *Sx*: HY9350_c0_g2_i1; and *Sy*: TBN21745_c0_g1_i1 (Figure 1). Therefore, these genes are the most appropriate to be considered as S alleles.

### Common Features With S-RNases

All known S-RNases are included in the T2 RNase protein family. T2 RNase contains two conserved amino acid sequences, CAS I and CAS II, which include two histidine residues, His46 and His109, essential for RNase activity (Kawata et al., 1990; Parry et al., 1997; MacIntosh, 2011). It was confirmed that the two conserved histidine residues were found in all the Sanger-sequenced genes in this study, suggesting that they have RNase activities. The intron was inserted at the same position in the non-conserved HV region among the T2 RNases (Supplementary Figure 2), which is identical to all S-RNases, with the exception of the *Prunus* in Rosaceae with additional introns in the first exon (Matsumoto and Tao, 2016). While S-RNase proteins identified in Rosaceae, Solanaceae, and Plantaginaceae have relatively basic pI, that of T2 RNases in classes I and II is acidic, with medians of 5.04, 5.90, and 8.56 and 9.18 in classes I, II, and III (non-S-RNase and S-RNase), respectively (Ramanauskas and Igic, 2017). Our data indicated the basic range of predicted pI values in class III sequences (Supplementary Figure 1). The functional causes of association of protein pI values are unclear, but pI appears to significantly correlate to S-RNase function (Ramanauskas and Igic, 2017). Thus, the shared feature of basic pI for citrus class III amino acid sequences fundamentally supports the hypothesis that these sequences are involved in the SI reaction.

S-RNases show pistil-specific expression. The expression analysis in different tissues revealed that HY8523 in LPSC3 was expressed not only in female organs but also in male organs (anther and pollen) (Figure 3). Gene expression of the female *S* determinant in GSI should be limited to the female organ. In addition, HY8523a is truncated, probably owing to a deletion that causes a frame shift, resulting in a shortened peptide. This sequence is not functional after duplication of the original gene. HY8523 is included in LPSC3, with Ciclev10003955m.g and Ciclev10003503m.g in the clementine genome (1.0), clementine0.9_033801m, clementine0.9_028427m, and clementine0.9_027797m in the clementine genome (0.9), and exist as tandem in each genome. Thus, it is concluded that they are not suitable as *S* determinants.

### A *T2 RNase* Downregulated in HYSC Style Tissue Possibly Associated With HYSI to HYSC Conversion

Excluding LPSCs, there were two ‘Hyuganatsu' genes in class III, HY11692 and HY9350. Quantitative gene expression analysis comparing HYSI and HYSC revealed that HY9350 was downregulated in HYSC by DEG analysis, which was further validated by qPCR (Figure 2; Supplementary Table 4). As Hyuganatsu transformation is difficult owing to its woody nature, a transgenic experiment to obtain direct evidence for the involvement of RNases in SI is impossible in Hyuganatsu, unlike in *Petunia* and *Nicotiana* as reported in other studies (Lee et al., 1994; Murfett et al., 1994). However, as HYSC is a bud sport of HYSI, mutation of a tiny part of the ‘Hyuganatsu' genome can be expected to cause a conversion from SI to SC. Thus, downregulation of the T2 RNase gene in the style tissue in HYSC strongly implies the association of that gene with the SI reaction. Moreover, low transcription levels of *S-RNase* attributed to the breakdown of SI have been reported in several *Prunus* species (Watari et al., 2007; Hanada et al., 2009; i Mart et al., 2010).

### *T2 RNase*s Showed Co-segregation With *S* Haplotypes

According to the report of Kim et al. (2011) and Zhou et al. (2018), *S* haplotypes of HYSI and TBN were assigned as *S1Sx* and *S1Sy*, respectively. As they have *S1* haplotypes in common, their status regarding cross-compatibility is semi-compatible (de Nettancourt, 1977). Segregation of the *S* haplotype in semi-compatible pollination does not follow the Mendelian law of segregation owing to self-incompatible reactions, and reciprocal crosses between semi-compatible partners lead to different *S* haplotypes in the progenies (Figure 4) (de Nettancourt, 1977). If a certain gene is an *S* gene or tightly linked to it, it would co-segregate with the *S* haplotype. This abnormal inheritance associated with GSI has been reported in some *Prunus* species, which show S-RNase-based GSI (Bošković et al., 1997; Choi et al., 2002).

From our results, each T2 RNase except for HY8523 was a heterozygotic allele because several negative individuals were found in the progeny populations for each T2 RNase. According to the segregation pattern of T2 RNases in the two populations, HY11692, HY9350, and TBN21745 were located at the same locus and corresponded to the *S* haplotype inheritance pattern, while inheritance of other genes was independent of the *S* haplotype inheritance manner (Figure 4). Thus, it can be considered that HY11692, HY9350, and TBN21745 correspond to *S1, Sx*, and *Sy* inheritance, respectively. This suggests that they are either *S* genes or, strictly speaking, genes closely linked to the *S* locus. Coupled with other evidence obtained in this study, they are most likely *S* genes. HY11692, the shared *S1* haplotype, was supported by the fact that a sequence (TBN9676), which was identical to HY11692, was found in TBN by RNA-seq analysis (Figure 1).

As HY16290 and TBN10176 were inherited with 3:1 and 1:1 segregation in both reciprocal crosses, their inheritance followed predicted Mendelian segregation. In addition, neither were located on the same locus as others, indicating that they are located on different chromosome(s) than the *S* haplotype. HY8523 is homozygous in at least one parent. As the *S* haplotype can generally not be homozygous, HY8523 is absolutely unrelated to the *S* locus and gene.

### Identity Between Isolated *T2 RNase*s, *S-RNase*s Identified in Chinese Citrus, and *S* Haplotypes Previously Determined in Japanese Citrus

Recently, it was demonstrated that the SI system is regulated by S-RNase in Chinese citrus species (Liang et al., 2020), and 15 *S-RNases* were identified. In this study, our results strongly imply that *T2 RNases* are involved in SI in Japanese citrus species. Kim et al. (2011) and Zhou et al. (2018) determined *S1, S2, S4*, and *S5* haplotypes for some Japanese citrus varieties, including HY, HSK, TBN, BAN, and SWS used in this study. As our data showed consistent correspondence between *S* haplotypes and *T2 RNase*s isolated in this study, suggesting that they are likely *S-RNases* (Figure 1), we assessed the homology of the sequences between six *T2 RNase*s and 15 *S-RNase*s determined by Liang et al. (2020). The results showed that three *T2 RNase*s were identical to the reported *S-RNase*s. Therefore, we propose that the three non-redundant *T2 RNases* are named *S15* to *S17 S-RNases*, and the *S* genotype of Japanese citrus cultivars should be relabeled based on the molecular types (Table 6).

Tachibana [*C. tachibana* (Makino) Tanaka] and Shiikwasha (*C. depressa* Hayata) are the only indigenous citrus species in Japan (Deng et al., 2020). During prehistoric and historical ages, several citrus types have been introduced, or naturally migrated to Japan, or occurred as natural hybrids (Omura and Shimada, 2016). Therefore, it is quite reasonable that Japanese citrus species share these genes with Chinese citrus. Liang et al. (2020) postulated the evolutionary history of citrus SC species considering the possession of *Sm*-RNase, which represents a self-compatible trait. Moreover, Zhou et al. (2018) speculated the establishment of some Japanese citrus cultivars focusing on the *S4* allele distribution in Japanese citrus varieties. Knowledge of the *S* allele constitution of the cultivars would be important not only as a genetic marker for growers' planning of new orchards and breeders' breeding programs, but also as a utility tool for researchers to trace the history of evolution, migration, and establishment of *Citrus* species. The universal nomenclature system for *Citrus* SI should be developed to avoid confusion about redundant *S* haplotypes among citrus varieties from different locations.

## Data Availability Statement

The datasets presented in this study can be found in online repositories. The names of the repository/repositories and accession number(s) can be found below: https://www.ddbj.nig.ac.jp/, PRJDB10130 (https://ddbj.nig.ac.jp/BPSearch) and LC575202–LC575209 (https://getentry.ddbj.nig.ac.jp/).

## Author Contributions

CH conceived the project, conducted experiments, and wrote the manuscript. KU performed RNA-seq library construction and data acquisition. MA and SI conducted PCR and electrophoresis in inheritance analysis. QY helped with qPCR analysis and revised the manuscript. FG supervised the project and revised the manuscript. TT supervised the project. All authors contributed to the article and approved the manuscript before submission.

## Conflict of Interest

The authors declare that the research was conducted in the absence of any commercial or financial relationships that could be construed as a potential conflict of interest.
