# Supplementary material for: Association of T2/S-RNase With Self-Incompatibility of Japanese Citrus Accessions Examined by Transcriptomic, Phylogenetic, and Genetic Approaches
Source: Front Plant Sci. 2021 Feb 12;12:638321. doi: 10.3389/fpls.2021.638321 (PMC7909312; doi:10.3389/fpls.2021.638321)
Supplement: Supplementary file 1 [file Data_Sheet_1.pdf]

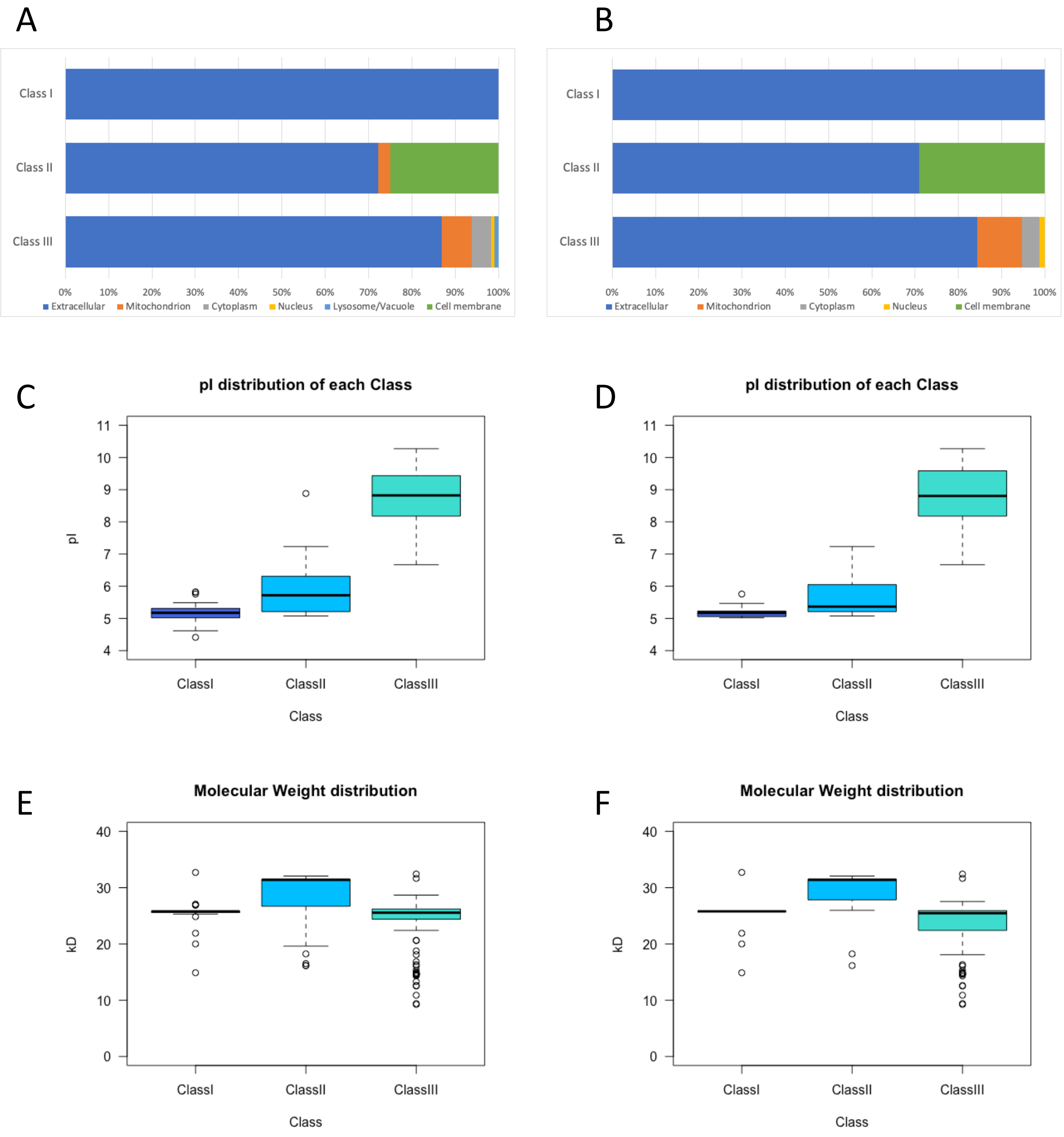

Fig. S1. Proportion of predicted subcellular localization (A and B), pI distribution (C and D), and molecular weight distribution (E and F) of T2 RNases for different classes in the phylogenetic tree (Fig. 1). A, C, and E: result from whole data set used for phylogenetic analysis. B, D, and F: result from a subset including only citrus T2 RNase data.

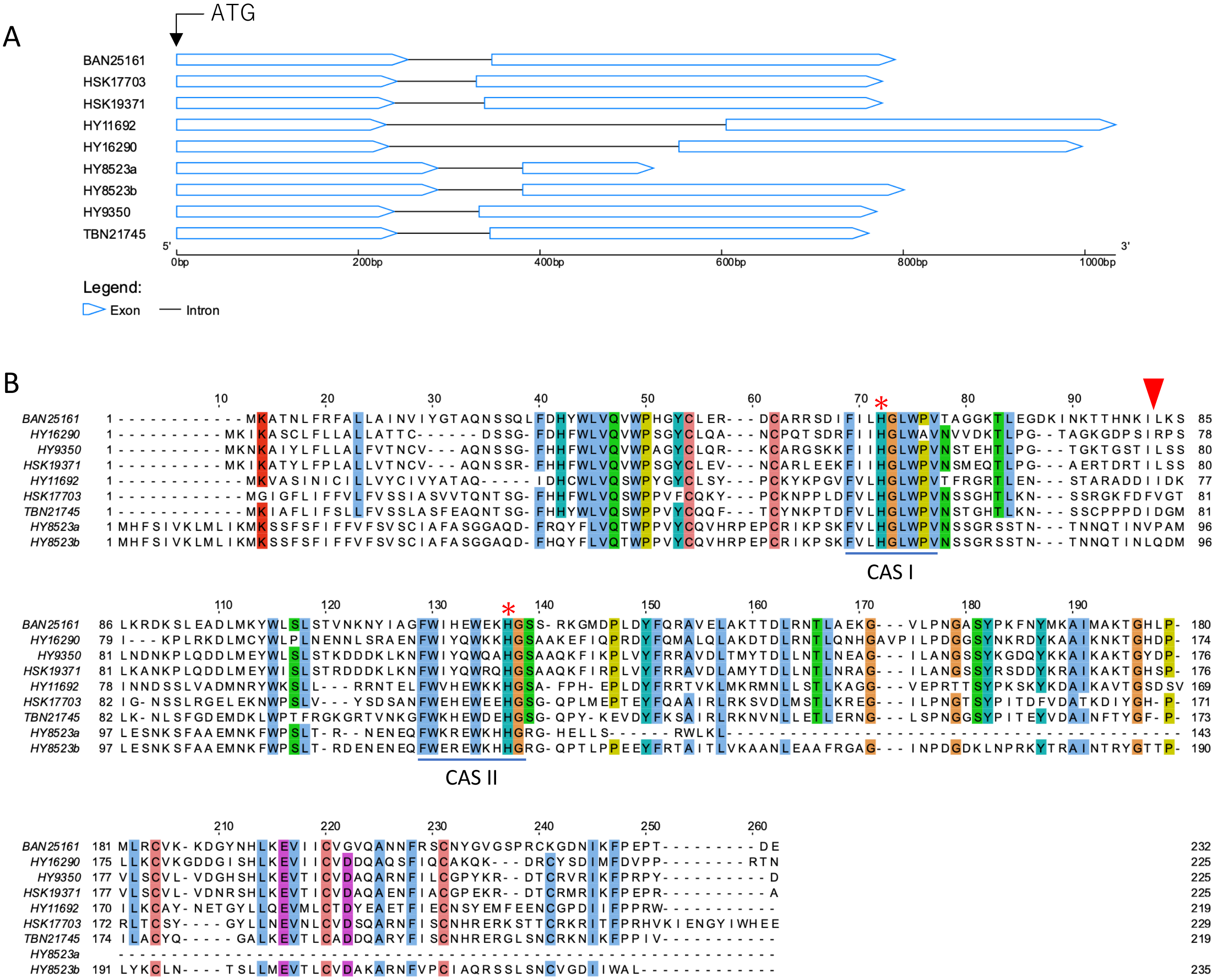

Fig S2. Genomic structure of class III T2 RNases. (A) Overview of genomic sequences of T2 RNases. Exons and intron were illustrated. (B) Alignment of amino acid sequence. Sites with over 70 % identity were colored. Intron position was indicated by the red arrowhead. The two conserved regions (CAS I and II) were indicated below the sequences. Two histidine residues essential for RNase activities in CAS I and II were pointed by asterisks.
